# Chemical and functional properties of cassava starch, durum wheat semolina flour, and their blends

**DOI:** 10.1002/fsn3.83

**Published:** 2014-01-20

**Authors:** Olufunmilola O Oladunmoye, Ogugua C Aworh, Bussie Maziya-Dixon, Ochuko L Erukainure, Gloria N Elemo

**Affiliations:** 1 Food Technology Department, Federal Institute of Industrial ResearchOshodi, Lagos, Nigeria; 2 Department of Food Technology, University of IbadanIbadan, Nigeria; 3 Crop Utilization Unit, International Institute of Tropical Agriculture (IITA)Ibadan, Nigeria

**Keywords:** Cassava starch, durum wheat semolina, flour blends, functional properties

## Abstract

High-quality cassava starch (HQCS) produced from high-yielding low-cyanide improved cassava variety, TMS 30572, was mixed with durum wheat semolina (DWS) on a replacement basis to produce flour samples containing 0, 20, 30, 50, 70, and 100% cassava starch. They were analyzed for chemical composition (proximate, amylose, free sugars, starch, wet gluten, and cyanide) and functional properties (pasting, swelling power, solubility, water absorption, water binding, starch damage, diastatic and *α*-amylase activity, dough mixing, and stability). Protein, carbohydrate, fat, and ash of flour samples ranged from 0.75–12.31%, 70.87–87.80%, 0.95–4.41%, and 0.12–0.83%, respectively. Cyanide levels in all the flour samples were less than 0.1 ppm. Amylose content varied between 19.49% for cassava and 28.19% for wheat, correlating significantly with protein (*r* = 0.95, *P* = 0.004) and ash contents (*r* = 0.92, *P* = 0.01) at 5%. DWS and HQCS had similar pasting temperatures (50.2–53°C), while other pasting properties increased with increasing levels of HQCS. Dough mixing stability of samples decreased with increasing levels of HQCS. All the flour samples had *α*-amylase activity greater than 200. Both HQCS and DWS compare favorably well in swelling power (7.80–9.01%); but the solubility of wheat starch doubled that of cassava. Starch damage varied between 3.3 and 7.2 AACC for semolina and starch, with the latter having higher absorption rate (97%), and the former, higher absorption speed (67 sec). Results obtained showed positive insight into cassava–wheat blend characteristics. Data thus generated provide additional opportunities of exploiting cassava utilization and hence boost its value–addition potentials for product development.

## Introduction

Cassava (*Manihot esculenta*) roots are largely cultivated in tropical countries. It has been earmarked as the crop that can spur rural industrial development and raise income for producers, processors, and traders (Echebiri and Edaba 2008). Cassava is the chief source of dietary food energy for the majority of people living in the lowland tropics, and much of the subhumid tropics of West and Central Africa. The biological characteristics of cassava, its ability to survive after cultivation, and the viability of its cuttings have contributed greatly to its spread (Lebot 2009).

Cassava has been viewed as a means of attaining household food security and increasing food availability (Lebot 2009). Low-cyanide variety (sweet cassava) was observed to meet these criteria and was thus suggested to be a good substitute for wheat flour in imported products. The adoption of high-yielding varieties and the resulting increase in yield have shifted the problem of the cassava sector from supply to demand issues, such as finding new uses and markets (Echebiri and Edaba 2008). This has led to intensive research on the use of its flour/starch in composite with wheat, for the development of bread and other bakery products, pastries, and confectioneries, and most recently, pasta products (Nweke et al. 2002; Oladunmoye et al. 2004; FIIRO 2006; Nwabueze and Anoruoh 2009).

However, certain properties of cassava flour and starch, such as physical, chemical, physicochemical, pasting, and thermal parameters are important for their being useful in food industries. More so, some functional characteristics have been reportedly correlated with certain key qualities of the products produced from such flours (Ponzio et al. 2008; Linlaud et al. 2009). Granulation characteristics of milled flours affect the rate of hydration and swelling capacity during processing (Hatcher et al. 2009); color determines visual appearance and eye appeal of finished product (MacDougall 2002); while water-binding and absorption capacities, swelling power, and solubility have a bearing on the carbohydrate quality and affect viscosity and gelling ability of flour/starch (Niba et al. 2001; Oladunmoye et al. 2004). Therefore, with the increasing interest in the use of cassava flour and starch in food product development, the availability of their chemical, functional, and pasting properties would lend itself as a processing protocol for the development of various value-addition food products. This study is aimed at reporting some chemical and functional properties of cassava starch, durum wheat semolina (DWS), and their blends.

## Materials and Methods

### Materials

High-yielding, low-cyanide cassava roots of improved cultivar TMS 30572 were obtained from the International Institute of Tropical Agriculture (IITA), Ibadan, Nigeria, and processed into high-quality starch (HQCS) within 24 h according to standard procedures developed and adopted by FIIRO (FIIRO 2006) at the pilot plant of the Federal Institute of Industrial Research, Oshodi (FIIRO), Lagos, Nigeria. DWS was obtained from flour mills of Nigeria, Lagos, Nigeria. These were kept dry in a refrigerator at 4°C during the course of this study.

### Sample preparation

Flour samples and blends were prepared on a replacement basis of DWS with HQCS to obtain 0, 20, 30, 50, 70, and 100% replacement with cassava starch. One kilogram of each sample/blend was weighed out, mixed thoroughly, and packaged in moisture-proof Nasco (Atkinson, WI) whirl-pak (180z.1532 ML Plain; ISO 9001 certified) and refrigerated at 4°C for further analysis.

### Physical properties

Granulation characteristics of HQCS and DWS were determined using a Retsch AS 200 basic mechanical shaker at amplitude 80 (ASTM International-Standards Worldwide 2006). Color was measured using Color Tec-PCM, (model SN 3000421; http://www.color-tec.com), operating on the CIE (Commission Internationale de l'Eclairage) color scheme, giving values expressed on the *L*,*a*,*b*, tristimulus scale (AOAC 2006).

### Proximate composition

Moisture content was determined using an air oven (model 655F; Fisher Scientific Co., Suwanee, GA) maintained at 105–176°C for 16–18 h, dried to constant weight (AACC 2005). Ash content was determined using a muffle furnace (model 186A; Fisher Scientific Co.) maintained at 600°C for 6 h (AOAC 2006). Crude protein was determined using the Kjeldahl method (Boric Acid Modification, Kjeltec 2300) (AACC 2005). Crude fat was determined using AACC 2005, Method 30-25.01 (Soxtec System HT2, Fisher Scientific Co.). Carbohydrate was estimated by difference and energy content calculated using Atwaters' factors.

### Chemical and functional characteristics

Starch and sugar contents were determined using the AOAC (2006) method in which 0.020 g finely ground sample was weighed into centrifuge tubes, wetted with 1 mL of ethanol, 2 mL of distilled water, followed by 10 mL hot ethanol. The mixture was vortexed and centrifuged using a Sorvall centrifuge (model GLC-1; Ivan Sorvall Inc., Newtown, CT) at 2000 rpm for 10 min. The supernatant was collected and used for free sugar analysis, while the residue was used for starch analysis.

Diastatic activity (maltose figure) and *α*-amylase activity (falling number) were determined using the AACC (2005) method.

Amylose/amylopectin contents of starch were determined using the total starch assay procedure (AACC 2005).

Cyanogenic potential was determined using the automated enzymic method developed by Rao and Hahn (1984) as modified by Bokanga (1994).

Water absorption and water-binding capacities were determined using AOAC (2006) methods. Starch damage was measured with the AACC (2005) method, while swelling power and solubility was determined by the modified method described by Riley et al. (2006).

### Pasting properties and mixing stability

Pasting properties were determined by an adaptation of the AACC (2005) using a Rapid Visco Analyzer 3 C (RVA, model 3C; Newport Scientific Pty Ltd, Sydney, NSW, Australia). The heating and cooling were at a constant rate of 11.25°C/min. Peak viscosity, holding strength, breakdown, final viscosity, set back, peak time, and pasting temperature were recorded with the aid of a computer (Thermocline for Windows Software; Newport Scientific). Mixing properties and stability was determined using the Brabender Extensograph (AACC 2005).

### Statistical analyses

All analyses were carried out in triplicate, unless otherwise stated. Statistical significance was established using one-way analysis of variance (ANOVA), and data were reported as mean ± standard deviation. Mean comparison and separation was done using Friedman's *t*-test (*P* < 0.05). Statistical analysis was carried out using the SAS 9.2 (http//www.sas.com/software/sas9/) statistical package.

## Results and Discussion

**Figure 1:**
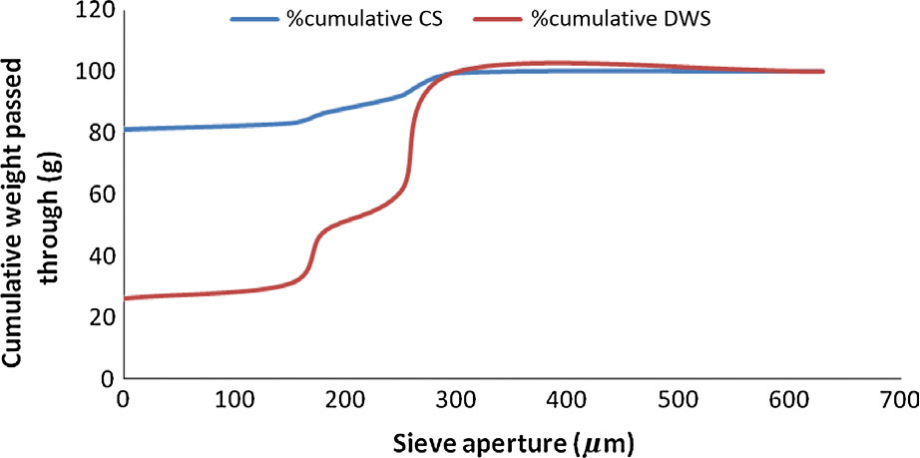
Particle size distribution of durum wheat semolina and cassava starch.

As shown in Figure 1, durum semolina used in this study was coarse and of medium granulation; the main mass fraction was between 250 and 300 *μ*m (38.8%). On the other hand, cassava starch used in this study had a finer particle size distribution (on milling) compared to wheat semolina, with the main mass fraction found to be <150 *μ*m (80.6%). Particle size distribution of milled flours affects the rate of hydration during processing, as very fine (<180 *μ*m) particle-sized flours have greater tendency of absorbing more water during hydration (Hatcher et al. 2009). Hou and Kruk (1998) reported that large particle flours required a longer time for water to incorporate and tend to form larger dough lumps. Tian et al. (1991) suggested that small granules have higher solubility and hence enhanced water absorption capacity, which have positive implications for functionality of flour during processing, often create more cohesion in most baking systems. Large granules, on the other hand, would be insufficiently hydrated. Optimum dough mixing would thus require fine and evenly distributed particle size flours. The particle size of the flours used in this study was below 300 *μ*m and was therefore easily hydrated.

**Figure 2:**
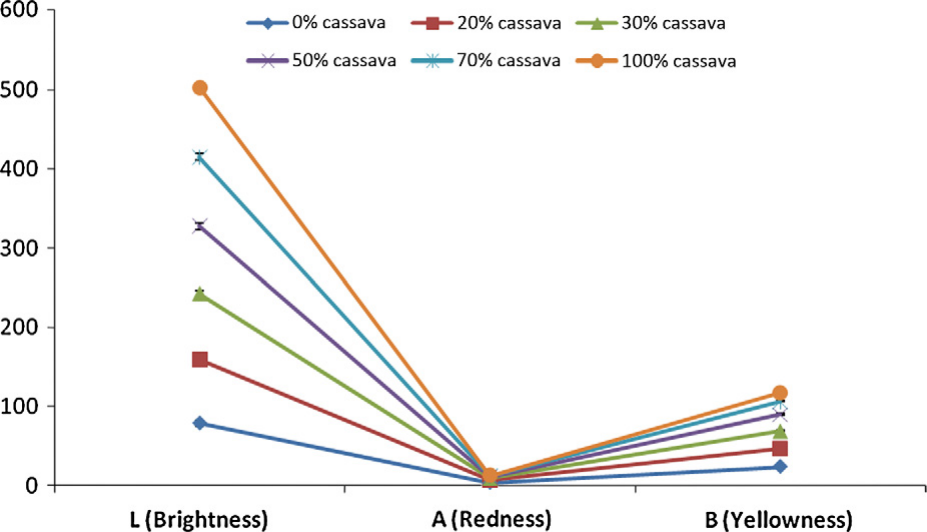
Brightness, redness, and yellowness of cassava starch, durum wheat semolina, and their blends.

Color of flour blends showed increasing brightness (*L*), reducing redness (*a*), and reducing yellowness (*b*) as white cassava starch was increasingly incorporated into the amber-colored DWS (Fig. 2).

The moisture content of all flour samples ranged between 10.38% for cassava starch and 11.58% for DWS, which is within the range acceptable for effective flour storage (Hayma 2003). As shown in Table 1, protein and ash contents of the flours varied between 0.75–12.31% and 0.12–0.83%; cassava starch having the least and DWS, the highest in respective order. Fat, ash, and protein contents reduced as level of wheat replacement increased. The very low protein value obtained for cassava starch is expected as cassava roots reportedly have 1.4 g protein and 0.6 g ash per 100 g edible cassava portion (Benders' Dictionary). On the other hand, wheat semolina had a protein level of 12.31% and an ash content of 0.83%, the former being as a result of its proteinous gluten matrix (Manthey and Schorno 2002). Carbohydrate content of flour blends increased as level of wheat replacement increased. There was a 6.61% increase in carbohydrate content at 30% replacement with cassava starch. This is because cassava is a starchy staple and a good source of carbohydrate (Lebot 2009).

**Table 1:** Proximate composition of cassava starch, durum wheat semolina, and their blends.

Flour containing	Moisture (%)	Fat (%)	Protein (%)	Ash (%)	Carbohydrate (%)	Energy^1^ (kcal/100 g)
100% Durum wheat semolina	11.6 ± 0.01^a^	4.4 ± 0.02^a^	12.3 ± 0.04^a^	0.8 ± 0.00^a^	70.9 ± 0.01^f^	372.4 ± 0.09^a^
20% Cassava starch	11.5 ± 0.01^a^	3.0 ± 0.02^b^	9.9 ± 0.03^b^	0.7 ± 0.01^b^	74.8 ± 0.04^e^	365.9 ± 0.10^b^
30% Cassava starch	11.5 ± 0.02^a^	2.9 ± 0.01^c^	8.5 ± 0.03^c^	0.7 ± 0.00^c^	76.4 ± 0.02^d^	365.9 ± 0.06^b^
50% Cassava starch	11.3 ± 0.01^b^	2.5 ± 0.03^d^	6.2 ± 0.06^d^	0.6 ± 0.01^d^	79.5 ± 0.05^c^	365.2 ± 0.19^c^
70% Cassava starch	11.3 ± 0.08^b^	2.2 ± 0.00^e^	4.6 ± 0.02^e^	0.4 ± 0.01^e^	81.5 ± 0.03^b^	364.0 ± 0.02^d^
100% Cassava starch	10.4 ± 0.08^c^	1.0 ± 0.00^f^	0.8 ± 0.13^f^	0.1 ± 0.00^f^	87.8 ± 0.13^a^	362.8 ± 0.01^e^
LSD	0.115	0.038	0.151	0.0168	0.148	0.239

Means with different superscripts within a column are significantly different at *P* < 0.05.

LSD, Fisher's least significant difference.

1 Values calculated using Atwaters' factors.

Amylose fraction in the starch component of the blends varied between 19.49% for cassava and 28.19% for wheat, though the amylose: amylopectin ratio was maintained at 0.3–0.4 in all the flour samples (Table 2). Similar amylose levels were reported by Kim and Wiesenborn (1996) for Mainechip potato starch (22.7%) and the commercial food grade potato starch (20.0%) obtained from Avebe Company, Veendam, Netherlands. The lower amylose content of HQCS compared with that of DWS makes it an ideal choice for higher digestibility (Riley et al. 2006). There was a strong and significant correlation of amylose level with protein (*r* = 0.95, *P* = 0.004) and ash contents (*r* = 0.92, *P* = 0.01) of flour samples; though its negative correlation with swelling power was not significant (*r* = −0.71, *P* = 0.11) at 5%.

**Table 2:** Chemical characteristics of cassava starch, durum wheat semolina, and their blends.

Flour sample containing	Starch (%)	Starch as amylose (%)	Cyanide (ppm)	Gluten	Sugar (%)
100% Durum wheat semolina	60.6 ± 1.06^f^	28.2 ± 0.39^a^	0.00 ± 0.0^c^	27.2 ± 0.14^a^	1.8 ± 0.38^bc^
20% Cassava starch	63.3 ± 0.67^e^	24.3 ± 0.62^b^	0.02 ± 0.0^b^	23.7 ± 0.71^b^	2.2 ± 0.02^a^
30% Cassava starch	71.3 ± 0.63^d^	24.4 ± 0.03^b^	0.02 ± 0.0^b^	18.5 ± 0.28^c^	2.2 ± 0.02^a^
50% Cassava starch	75.3 ± 0.02^c^	22.0 ± 0.54^c^	0.02 ± 0.0^b^	10.7 ± 0.57^d^	1.6 ± 0.00^c^
70% Cassava starch	80.3 ± 0.02^b^	19.9 ± 0.28^d^	0.02 ± 0.0^b^	1.2 ± 0.14^e^	2.1 ± 0.00^ab^
100% Cassava starch	84.5 ± 0.48^a^	19.5 ± 0.07^d^	0.05 ± 0.0^a^	0.0 ± 0.00^f^	1.5 ± 0.01^c^
CV	0.83	1.69	0	2.92	8.32
LSD	1.48	0.96	0	0.97	0.39

Means with different superscripts within a column are significantly different at *P* < 0.05. ppm, parts per million.

As shown in Table 3, diastatic activity (maltose figure) and *α*-amylase activity (falling number) of cassava starch (96 mg/10 g and 260) was lower than that of wheat semolina (244 mg/10 g and 702). At 20% replacement, there was about 12.7% and 29.1% reduction in diastatic activity and amylase activity values, respectively. This indicates a very moderate *α*-amylase activity in TMS 30572 cassava starch. Falling number value ranged from 260 sec for 100% cassava starch, to 702 sec for 100% wheat semolina. These high values (>200) suggest that bakery and pasta products made with these flour blends would probably exhibit some acceptable characteristics. However, Mühlenchemie (2007), while responding to questions raised on flour standardization, noted that for a high falling number though desirable to achieve certain specified flour properties, the bake-ability of the flour and the attributes of the end product need to be given adequate consideration.

**Table 3:** Functional properties of cassava starch, durum wheat semolina, and their blends.

Parameters	100% Durum wheat semolina	20% Cassava starch	30% Cassava starch	50% Cassava starch	70% Cassava starch	100% Cassava starch	LSD
Diastatic activity (mg/10 g)	244.0 ± 1.41^e^	213.0 ± 1.41^d^	182.5 ± 2.12^c^	177.5 ± 2.12^b^	140.5 ± 2.12^a^	96.0 ± 2.83^f^	5.04
*α*-Amylase activity (sec)	702.0 + 24.0^a^	498.0 + 14.14^b^	483.5 + 44.55^bc^	430.0 + 15.56 ^cd^	410.0 + 7.07^d^	260.0 + 2.83^e^	55.28
Swelling power (%)	7.8 ± 0.04^c^	7.5 ± 0.04^d^	7.4 ± 0.03^d^	7.9 ± 0.03^c^	8.3 ± 0.03^b^	9.0 ± 0.17^a^	0.18
Solubility (%)	4.4 ± 0.04^a^	3.9 ± 0.01^b^	3.8 ± 0.01^c^	3.8 ± 0.02^bc^	3.4 ± 0.03^d^	2.2 ± 0.03^e^	0.06
WAC (%)	93.0 ± 0.37^e^	97.0 ± 0.23^d^	98.6 ± 0.37^d^	103.2 ± 1.61^c^	126.6 ± 1.25^b^	164.7 ± 1.15^a^	2.41
WBC (%)	69.6 ± 0.21^f^	90.6 ± 0.89^e^	94.8 ± 1.04^d^	105.7 ± 0.28^c^	117.9 ± 1.10^b^	147.5 ± 1.22^a^	2.17
Starch damage AACC (%)	3.3 ± 0.01^f^	4.5 ± 0.06^e^	5.5 ± 0.07^d^	6.3 ± 0.03^c^	6.9 ± 0.04^b^	7.2 ± 0.04^a^	0.11
Abs rate (%)	91.4 ± 0.14^f^	93.5 ± 0.04^e^	95.0 ± 0.21^d^	96.1 ± 0.03^c^	96.8 ± 0.03^b^	97.1 ± 0.03^a^	0.26
Abs speed (sec)	67.0 ± 1.41^a^	52.0 ± 0.00^b^	39.0 ± 1.41^c^	30.0 ± 1.41^d^	24.0 ± 1.41^e^	28.0 ± 0.00^d^	2.83

Means with different superscripts within a column are significantly different at *P* < 0.05. WAC, water absorption capacity; WBC, water-binding capacity; Abs, absorption.

Gluten content was observed to reduce as levels of wheat replacement increased, indicating increased dilution of its gluten matrix. The efficacy of cyanide removal from cassava roots during starch preparation using the FIIRO detoxification technique (FIIRO 2006) is shown in its level becoming reduced to 0.05 ppm from the reported 14.20 ppm for TMS30572 cassava cultivars (Dixon et al. 2010). This is a 99.6% reduction via processing through grating operation, thus confirming the report of Dufour (2007) who reported a range of 93 to >99% reduction using similar processing methods. This method of cassava root disintegration is gaining ground also for flour production, compared to the chipping method (FIIRO 2006).

There was a gradual decline in swelling power and solubility of wheat semolina as it was replaced with cassava starch up to 30%; further replacement resulted in increased swelling power from 7.89 g/g at the 50% level to 8.30 g/g at 70% and 9.01 g/g at 100%. This probably indicates the level at which the swelling tendency of cassava starch overpowers that of wheat starch (Table 3). On the other hand, solubility which increased to 3.81 at 50% replacement thereafter became reduced with further incorporation of cassava starch. A similar trend was observed in earlier reports and was attributed to the low-fat content and the weak internal organization within root and tuber starches. This is probably caused by the negatively charged phosphate ester groups within their starch granule (Kim and Wiesenborn 1996). This explains why water absorption capacities and water-binding capacities increased with increasing starch incorporation (Table 3). Table 3 further shows the extent to which the respective starches were damaged, indicating increase from 3.25% to 7.17% as cassava starch incorporation increased from 0% to 100%. This accounts for the increases observed in water absorption capacities as damaged starch is able to absorb four to five times more water than intact starch (Hatcher et al. 2009). This has, however, been associated with increased stickiness of cooked pasta. Absorption rate was likewise increased from 91.44% to 97.12%, though the speed at which this took place got gradually reduced from 67 sec, for DWS, to 28 sec for cassava starch. These results suggest that 50% cassava starch is the best level for replacement.

There was little or no variation in the pasting temperatures of cassava starch (50.25°C), DWS (50.18°C), and their blends (50.18–50.23°C) as shown in Table 4. This probably indicates some similarity between the two starches, despite being from root and tuber and cereal, respectively. However, higher values were obtained for potato starches (62.7–67.8°C). On the other hand, other pasting properties including peak viscosity (96.3 RVU for durum wheat; 364 RVU for cassava starch), holding strength (70.8–153.5 RVU), breakdown (25.5–210.8 RVU), and final viscosities (150.1–216 RVU) were generally much higher as cassava starch inclusion increased, while setback value and peak time reduce. Swelling power was positively correlated with peak viscosity (*r* = 0.85), but negatively with setback value (*r* = −0.027). This negative correlation was, however, not significant (*P* > 0.05).

**Table 4:** Pasting properties of cassava starch, durum wheat semolina, and their blends.

Flour containing	Peak viscosity (RVU)	Holding strength (RVU)	Break down (RVU)	Final viscosity (RVU)	Setback value (RVU)	Peak time (min)	Pasting temperature (^°^C)
100% Durum wheat semolina	96.3 ± 3.54^d^	70.8 ± 2.35^d^	25.5 ± 1.17^d^	150.1 ± 2.06^b^	72.3 ± 0.30^a^	5.3 ± 0.09^a^	50.2 ± 0.04^a^
20% Cassava starch	124.1 ± 4.01^c^	81.0 ± 5.06^bc^	43.1 ± 1.06^c^	144.0 ± 6.01^b^	63.0 ± 0.95^b^	5.3 ± 0.09^a^	50.2 ± 0.11^a^
30% Cassava starch	132.0 ± 2.77^c^	83.6 ± 1.94^bc^	48.5 ± 0.82^c^	141.1 ± 3.30^b^	57.5 ± 1.36^c^	5.0 ± 0.02^b^	50.2 ± 0.07^a^
50% Cassava starch	155.2 ± 2.35^b^	78.0 ± 1.00^c^	77.2 ± 1.36^b^	127.7 ± 0.12^c^	49.7 ± 0.88^d^	4.1 ± 0.09^c^	50.2 ± 0.04^a^
70% Cassava starch	134.2 ± 2.47^c^	86.1 ± 1.35^b^	48.0 ± 1.12^c^	144.3 ± 1.41^b^	58.1 ± 0.06^c^	5.0 ± 0.07^b^	50.2 ± 0.11^a^
100% Cassava starch	364.3 ± 12.49^a^	153.5 ± 3.54^a^	210.8 ± 8.96^a^	216.0 ± 6.66^a^	62.5 ± 3.13^b^	4.0 ± 0.04^c^	50.3 ± 0.14^a^
CV	3.48	3.14	5.03	2.62	2.42	1.55	0.18
LSD	14.27	7.08	9.29	9.87	3.65	0.18	0.22

Means with different superscripts within a column are significantly different at *P* < 0.05. RVU, Rapid Viscosity Unit.

**Figure 3:**
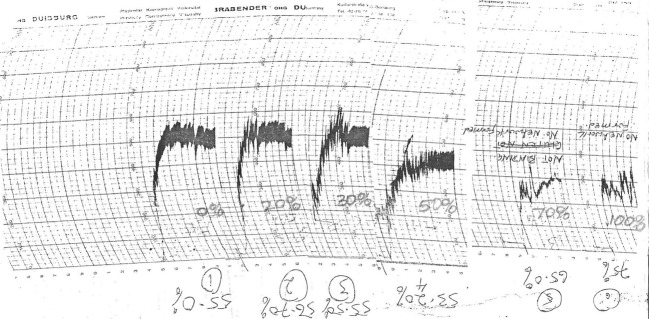
Farinogram curves at 0, 20, 30, 50, 70, and 100% substitution of durum wheat semolina with cassava starch.

Mixing stability of flour samples decreased as level of cassava inclusion increased (Fig. 3), showing that gluten constituent, on which dough extensibility and viscoelasticity depend, was being increasingly diluted. This accounts for the weak binding forces and unstable Farinogram curves recorded at 50, 70, and 100% substitution levels (Table 2, Fig. 3). This is because cassava starch, though exhibiting some viscoelastic properties, lacks gluten. A similar trend was observed by Gunathilake and Abeyrathne (2008) while incorporating coconut flour into noodle flour beyond 30%.

## Conclusion

This study revealed that HQCS obtained from TMS 30572 cassava variety exhibits certain properties that could complement DWS in pastry and pasta production. The gelatinization characteristics of HQCS, its swelling power, solubility properties, and low-amylose content offer some unique advantages that suggest its suitability, not only for pastry and pasta production but also in other food applications. Variations in the functional properties of the blends studied could be of significance in choosing wheat replacement level for different products. Hence, partial replacement of DWS with HQCS would yield desirable results. Available data could thus serve as a guiding protocol to the development of pastries, pasta, and other food products from a blend of HQCS and DWS.

## References

[b1] AACC (2005).

[b2] AOAC (2006).

[b3] ASTM International-Standards Worldwide (2006). http://www.astm.org/cgibin/SoftCart.exe/DATABASE.CART/REDLINE_PAGES/C136.htm?E+mystore.

[b4] Benders' Dictionary http//www.stu.edu.vn/index.php?r=site/downloadEbook&id=268.

[b5] Bokanga M (1994). Processing of cassava leaves for human consumption. Acta Hortic.

[b6] Dixon AGO, Okechucku RU, Akoroda MO, Ilona P, Ogbe F, Egesi CN (2010). Improved cassava variety handbook.

[b7] Dufour DL, Ortiz R, Nassar NMA (2007). Bitter cassava: toxicity and detoxification. Proceedings of the first international meeting on cassava breeding, biotechnology and ecology.

[b8] Echebiri RN, Edaba MFI (2008). Production and utilization of cassava in Nigeria: prospects for food security and infant nutrition. PAT.

[b9] FIIRO (2006). Cassava: production, processing and utilization in Nigeria.

[b10] Gunathilake KDPP, Abeyrathne YMRK (2008). Incorporation of coconut flour into wheat flour noodles and evaluation of its rheological, nutritional and sensory characteristics. J. Food Process Preservat.

[b11] Hatcher DW, Bello GG, Anderson MJ (2009). Flour particle size, starch damage, and alkali reagent: impact on uniaxial stress relaxation parameters of yellow alkaline noodles. Cereal Chem.

[b12] Hayma J (2003). The storage of tropical agricultural products.

[b13] Hou GQ, Kruk M (1998).

[b14] Kim YS, Wiesenborn DP (1996). Starch noodle quality and related to potato genotypes. J. Food Sci.

[b15] Lebot V, Lebot V (2009). Cassava: postharvest quality and marketing. Tropical root and tuber crops cassava, sweet potato, yams and aroids.

[b16] Linlaud NE, Puppo MC, Ferrero C (2009). Effect of hydrocolloids on water absorption of wheat flour and farinograph and textural characteristics of dough. Cereal Chem.

[b17] MacDougall D (2002). Colour in food: improving quality.

[b18] Manthey FA, Schorno AL (2002). Physical and cooking quality of Spaghetti made from whole wheat durum. Cereal Chem.

[b19] Mühlenchemie (2007). http://www.muehlenchemie.de/english/know-how/questions-and-anwers.html.

[b20] Niba LL, Bokanga MM, Jackson FL, Schlimme DS, Li BW (2001). Physicochemical properties and starch granular characteristics of flour from various *Manihot esculenta* (Cassava) genotypes. J. Food Sci.

[b21] Nwabueze UT, Anoruoh AG (2009). Clustering acceptance and hedonic responses to cassava noodles extruded from cassava mosaic disease-resistant varieties. Afr. J. Food Sci.

[b22] Nweke FI, Spencer DSC, John KL (2002). The cassava transformation; Africa's best kept secret.

[b23] Oladunmoye OO, Ozumba AU, Oluwole OB, Orishagbemi CO, Solomon HM, Olatunji O (2004). Development of process technology for cassava-based noodle products. J. Sci. Eng. Tech.

[b24] Ponzio NR, Puppo MC, Ferrero C (2008). Mixtures of two Argentinean wheat cultivars of different quality: a study on breadmaking performance. Cereal Chem.

[b25] Rao PO, Hahn SK (1984). An automated enzymatic assay for determining the cyanide content of cassava (*Manihot esculenta* Crantz) and cassava products. J. Sci. Food Agric.

[b26] Riley CK, Wheatley AO, Asemota HN (2006). Isolation and characterization of starches from eight Dioscorea alata cultivars grown in Jamaica. Afr. J. Biotechnol.

[b27] Tian Q, Streuli M, Saito H, Schlossman SF, Anderson P (1991). A polyadenylate binding protein localized to the granules of cytolytic lymphocytes induces DNA fragmentation in target cells. Cell.

